# A spontaneous paracecal herniation: a rare form of an uncommon case

**DOI:** 10.1093/jscr/rjad037

**Published:** 2023-02-06

**Authors:** Bright Anderson Mwanje, Melvin Gloria Nassaka, Andrew Marvin Kanyike

**Affiliations:** Department of Surgery, Mengo Hospital, Kampala, Uganda; Department of Surgery, Mengo Hospital, Kampala, Uganda; Department of Surgery, Mengo Hospital, Kampala, Uganda

**Keywords:** spontaneous, retroperitoneal hernia, paracecal hernia, rectal adenocarcinoma

## Abstract

Paracecal hernias are a rare form of internal hernias. This is a case of a spontaneous paracecal retroperitoneal hernia in an elderly patient. A 73-year-old male, a known patient of adenocarcinoma of the rectum, who had undergone neoadjuvant radiotherapy and chemotherapy a year ago, presented with a 2-day-history of exacerbated colicky peri-umbilical and right-sided non-radiating abdominal pain, with associated progressive abdominal distension, which were aggravated by feeding. A contrasted abdominal computed tomography scan showed features of small bowel obstruction. An exploratory laparotomy revealed herniation of gangrenous small bowel ~30 cm through a small, tight opening just below the base of the cecum. Resection and anastomosis and closure of the retroperitoneal pouch were done. Patient recovered and was discharged on the fourth post-operative day. Spontaneous retroperitoneal hernias can occur in elderly patients with additional risk factors like neoplasms, with a high risk of bowel ischemia, hence the urgent need for surgical intervention.

## INTRODUCTION

Internal hernias occur rarely, involving protrusion of intra-abdominal organs or mesentery into abnormal anatomic spaces [1]. They can be primary in the case of congenital defects like very large foramen of Winslow or secondary due to pathologic openings following surgery, trauma or infection [1]. Retroperitoneal hernias are the rarest form of internal hernia due to the multi-fascial layers and rigidity of the posterior peritoneum [2]. As first elucidated by Treiz way back in 1857, they usually occur in natural fossae, like the para-duodenal [3]. Most retroperitoneal hernias, reported in literature, followed peritoneal injury after surgery, either via laparoscopy or open nephrectomy [4–7]. In this case report, we present a rare occurrence of spontaneous paracecal hernia, a very rare occurrence of internal hernia.

## CASE REPORT

**Figure 1:**
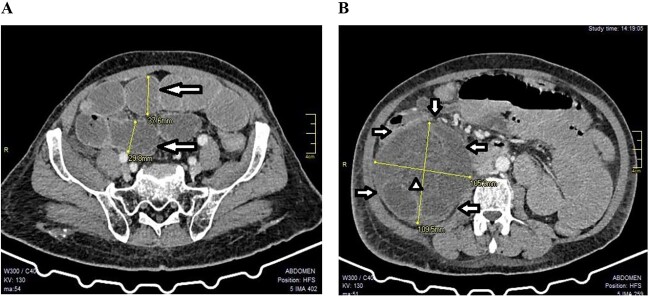
A CT scan showed features of intestinal obstruction around the paracecal site with dilated small bowel loops; (**A**) the various dilated small bowel loops (arrows) can be seen; (**B**) the hernia sac (arrows) with dilated bowel loops inside (arrow head) were seen around the cecum (axial images).

A 73-year-old male, a known patient of adenocarcinoma of the rectum for 1 year, who had undergone neoadjuvant radiotherapy and chemotherapy a year prior and got lost to follow-up, presented at our hospital with a 2-day-history of exacerbated colicky peri-umbilical and right-sided non-radiating abdominal pain, with associated progressive abdominal distension, aggravated by feeding. These were preceded by 2 weeks of difficulty in passing stool and gas (that could be temporarily relieved by the use of the laxative, bisacodyl), nausea and vomiting, initially bilious then overt fecal contents, loss of appetite and generalized body weakness. Two weeks before admission to our hospital, he had been managed nonoperatively for ‘appendicitis’ at a peripheral health facility without improvement, hence the referral. He had no history of any previous surgeries. Physical examination at admission was remarkable for a moderately distended abdomen with marked peri-umbilical and right iliac fossa tenderness, with reduced bowel sounds. On digital rectal exam, the rectum was empty with a tipped polypoid mass high up, ~8 cm from the anal verge. A contrasted abdominal computed tomography (CT) scan was done, which reported features of small bowel obstruction, rectal neoplasm with local metastases to the prostate and urinary bladder and mild pleural effusion with bilateral basal consolidation (Fig. 1).

Laboratory investigations performed at admission are presented in Table 1. A diagnosis of sub-acute small bowel obstruction was made, and management constituted intravenous crystalloids, antibiotics and analgesics; he was also put on Nil per Os with nasal gastric tube decompression and was scheduled for surgery the following day.

**Table 1:** Summary of laboratory investigation results on admission

Blood investigation	Normal range	Results
WBC count	4.00–10.00/Ul	9.78 mg/l
Absolute neutrophils	1.50–7.00	9.89
Absolute lymphocytes	1.00–3.70	0.53
Hemoglobin	12.0–18.0 g/dl	10.8 g/dl
Hematocrit	36–51%	31.9%
Platelets	150–400/Ul	183/Ul

**Figure 2:**
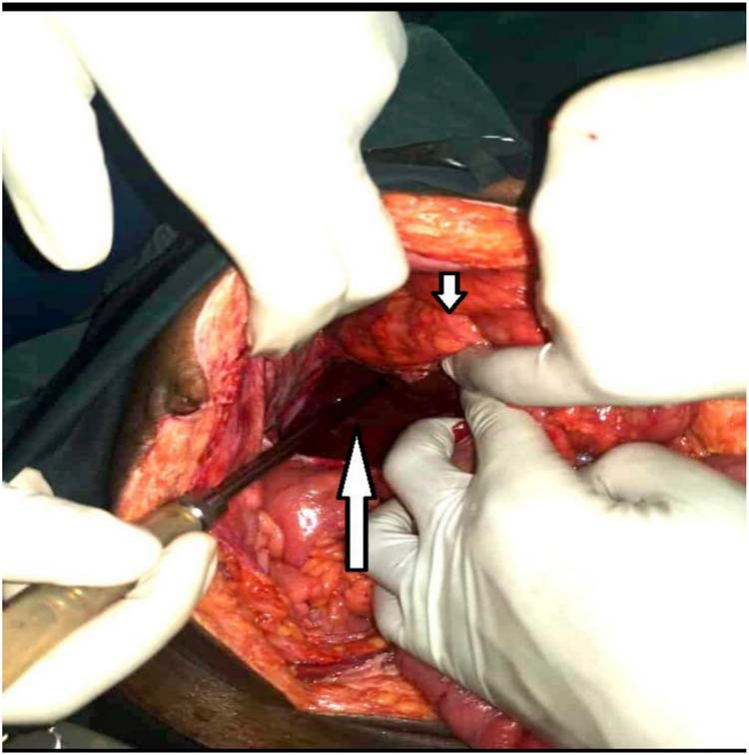
Large retroperitoneal paracecal pouch (long arrow) and the cecum above it (short arrow).

An exploratory laparotomy under general anesthesia was done. Intraoperatively, we found a large right retroperitoneal mass formed by a tuft of bowel that had herniated through a small tight (1.5 cm wide) opening just below the base of the cecum, creating a retroperitoneal pocket full of hemorrhagic fluid (~600 ml) and gangrenous ileum of ~30 cm, cecum and appendix. There was an associated ileal perforation ~15 cm from the ileocecal junction (Fig. 2). The gangrenous bowel loop was resected and an end-to-end, ileo-ascending, single-layered, hand-sewn anastomosis was done. The retroperitoneal pouch was lavaged with warm saline and was then closed over a tube drain. On inspection of the rest of the intra-abdominal contents, a rectal tumor was found with the dilated descending and sigmoid colon, but no extra-serosal, adjacent nor obvious distant organ metastasis. A pre-emptive diversion loop sigmoidostomy was done and the abdomen was closed in layers. Post-operatively: the patient was kept on intravenous piperacillin/tazobactam, metronidazole, morphine and paracetamol, kabiven and crystalloid fluids. Enteral feeds were gradually re-introduced, starting with clear fluids on the second post-operative day, and the patient was taught colostomy self-care. He recovered steadily without major post-operative complications and was discharged on Day 4 post-operatively through the Cancer Institute for re-evaluation for more cycles of neo-adjuvant chemotherapy before scheduling him for a low anterior resection.

We are currently following him up closely to avoid a repeat of the loss to follow-up to make the most of his chance at having the best possible outcome for the rectal cancer treatment.

## DISCUSSION

A retroperitoneal hernia is a form of internal herniation that occurs rarely and poses difficulty with diagnosis. Since the first description of such a hernia by Treitz in 1857, a few case reports, especially of the adulthood-acquired form, have been reported in the literature [8]. Unlike this patient, retroperitoneal hernias reported share common etiology following a tear in the posterior peritoneum because of trauma like lumbar fractures [9] and nephrectomy [4–7]. They could as well be caused by congenital malformations as seen in the pediatric population [7]**.** These hernias can be categorized according to the anatomical areas they occur into six groups—paraduodenal, pericecal, intersigmoid, transmesenteric, pelvic and supravesical and those through the foramen of Winslow. Furthermore, the pericecal can be sub-categorized into superior ileocecal recess, inferior ileocecal recess, paracolic sulcus and retrocecal recess [10]. The paraduodenal hernias are commonest that are thought to occur as a result of abnormal gut rotation due to fusion folds within the peritoneum [10].

Internal hernias rarely cause intestinal obstruction, accounting for only 4% of all cases of small bowel obstruction. However, they can be fatal with mortality up to 50% if managed untimely, making early diagnosis very vital in preventing gut ischemia [11] The incidence of retroperitoneal hernia is very low and without specific manifestation, therefore the suspicion index should be high with features of SBO following prior surgery [7]. Radiographical investigations are key in the initial evaluation and early support diagnosis of retroperitoneal hernia, but these are not without challenges. Osadchy and colleagues provide some characteristic descriptions of findings for a retroperitoneal hernia on a CT scan [12]**.** However, the peritoneal cavity and posterior peritoneal structure are complicated, and it is challenging to identify the abdominal ligaments, mesentery and peritoneum on the CT scan. The small intestine has relative freedom of movement, and its position is variable, making the pre-operative diagnosis even more difficult [1]**.**

Our patient had a paracecal hernia that was spontaneous, presenting with features of small bowel obstruction and gangrenous bowel. It is elucidated in literature that paracecal hernias could be as a result of tissue weakening due to age, high intra-abdominal pressure and retroperitoneal adhesions among other factors [13]. Most probably age could be pinpointed in our patient as a major risk factor for the herniation. It is also worth noting that he had a history of neoplasm with some cycles of chemotherapy. Intra-operatively, the colon cancer had not metastasized and cannot be wholly implicated. Elderly patients with intestinal malignancy presenting with features of bowel obstruction should ring a bell for possible internal herniation. This case brings to limelight the fact that a retroperitoneal hernia can develop spontaneously in adulthood, or possibly a congenital defect manifesting later in life under stressful conditions.

## CONCLUSION

We present a unique case in terms of the spontaneous nature of a paracecal hernia unlike those reported following trauma or surgery. Age and neoplastic processes may lead to tissue weakening and may cause protrusion of organs through otherwise strong tissues. Therefore, presentations of acute abdomen or features of intestinal obstruction among the elderly should ring a bell for internal hernias and warrant immediate surgical management.

## CONFLICT OF INTEREST STATEMENT

None declared.

## FUNDING

No funding was received for this case report.

## DATA AVAILABILITY

Data used in this case report is available upon appropriate request from the corresponding author.
